# Bilateral Rectus Sheath Block with Continuous Bupivacaine Infusions After Elective Open Gastrectomy: A Randomized Controlled Trial

**DOI:** 10.3390/medicina60121992

**Published:** 2024-12-02

**Authors:** Janis Opincans, Igors Ivanovs, Aleksejs Miscuks, Janis Pavulans, Katrina Deja Martinsone, Agris Rudzats, Zurabs Kecbaja, Olegs Gutnikovs, Aleksejs Kaminskis

**Affiliations:** 1 Department of Surgery, Riga East Clinical University Hospital, 1038 Riga, Latvia; igors.ivanovs@aslimnica.lv (I.I.); katrina.deja.martinsone@rsu.lv (K.D.M.); agris.rudzats@aslimnica.lv (A.R.); zurabs.kecbaja@aslimnica.lv (Z.K.); aleksejs.kaminskis@aslimnica.lv (A.K.); 2 Faculty of Medicine, University of Latvia, 1004 Riga, Latvia; 3 Faculty of Medicine, Riga Stradins University, 1007 Riga, Latvia

**Keywords:** multimodal analgesia, laparotomic gastrectomy, bupivacaine infusion, rectus sheath block, enhanced recovery

## Abstract

*Background and Objectives:* Multimodal analgesia has been shown to be effective in facilitating early postoperative gastrointestinal function and rehabilitation in patients undergoing open gastrectomy. We conducted a clinical trial to investigate the effectiveness of bilateral rectus sheath block (RSB) with continuous bupivacaine infusion in comparison with placebo following elective open gastrectomy. *Materials and Methods:* Patients indicated for elective open gastrectomy were screened, enrolled, and randomised between October 2021 and September 2023. The patients were randomised to either Group A (intervention—continuous bupivacaine) or Group B (control—NaCl saline). The primary outcome measures were the quantity of an opioid analgesic administered during the initial 72 h post-surgery and the level of postoperative pain as indicated by the visual analogue scale (VAS). Mann–Whitney U test was used for quantitative analysis while Pearson Chi-square was used for categorical variables. *Results:* A total of 60 patients completed the trial, with 30 patients in each of the two groups. Patients in Group A reported lower median VAS pain scores at all observed time points following surgery (*p* < 0.001). No patient in Group A required rescue opioid analgesia, although non-steroidal anti-inflammatory drugs were necessary during the initial 12 h postoperatively. Clinically, Group A patients had a significantly shorter time to first gas (*p* = 0.001), a shorter time to first bowel movement (*p* < 0.001), a shorter time to first out-of-bed activity (*p* < 0.001), and a shorter overall hospitalisation duration (*p* < 0.001) compared to Group B patients. *Conclusions:* Bilateral RSB with continuous bupivacaine infusion is effective in managing pain and can reduce the use of opioid analgesics in the postoperative period. Furthermore, it promotes early recovery, and a shorter hospital stay.

## 1. Introduction

A number of different analgesic techniques are used to provide post-operative pain relieve following major intra-abdominal surgeries. Although these techniques are generally regarded as highly effective, they are known to be associated with postoperative gastrointestinal dysfunction (POGD) and other medication-related adverse effects such as abdominal discomfort, constipation, and intestinal obstruction [1,2]. The aetiology of POGD is typically multifaceted, encompassing factors such as damage to intestinal barrier function, systemic inflammatory mediators, stress responses, opioid use, and abnormal abdominal distention [3].

Clinically, POGD has been associated with increased healthcare costs, including extended nursing care, supplementary radiological examinations, medication prescriptions, laboratory expenses, and allied health procedures. For instance, the administration of high doses of opioids, particularly to elderly patients, has been associated with delayed gastrointestinal mobilisation and an increased risk of prolonged hospital stays [3]. In a similar analysis from a nationwide retrospective audit in the United States [4] reported that 12.8% of all patients undergoing open gastrectomy experienced POGD and postoperative paralytic ileus. A single-centre prospective audit from New Zealand also observed that POGD increased the median cost of an inpatient stay for patients undergoing elective gastrointestinal and colorectal surgery by up to 71% [5].

To address these challenges, multimodal analgesia has been proposed as a superior approach to traditional analgesia. Multimodal analgesia is defined as a combination of different techniques, including the use of local or regional analgesia with general analgesia (such as non-steroidal anti-inflammatory drugs or opioids). A key advantage of multimodal analgesia is the reduction in the dosage and frequency of opioids administered, while concurrently decreasing the incidence of adverse effects associated with their use.

Among the several regional analgesia techniques used, regional blockage and epidural analgesia are the most preferred approaches. The rectus sheet block (RSB) represents a promising avenue for providing regional analgesia, particularly in the context of open (laparotomic) surgeries, including open gastrectomies. However, regional anaesthetic techniques remain constrained by several limitations, chief among which are the time-consuming nature of the procedure, the necessity for additional equipment and personnel, the lack of operational standards and procedures, and the consensus on optimal catheter insertion techniques. Despite the growing popularity of the RSB, these shortcomings are yet to be fully addressed [6,7].

Based on the evidence in the reported literature, we postulated that RSB with continuous infusion of bupivacaine, a local anaesthetic, following open gastrectomy could enhance postoperative gastrointestinal function and facilitate early recovery, while reducing complications due to decreased opioid use. Bupivacaine was selected as the preferred anaesthetic agent for this study, as it has a significantly longer duration of action than lidocaine, although with a delayed onset. Bupivacaine typically initiates its pharmacological effect within five minutes and persists for two to four hours, with a maximum recommended dose of 2 mg/kg. Lidocaine typically initiates its pharmacological effect in less than two minutes, with a duration of action extending to one to two hours. The maximum recommended dose is 5 mg/kg [8].

Accordingly, to test our hypothesis, we designed the present single-centre clinical trial with the objective of exploring the postoperative rehabilitation process of gastrointestinal function and the potential mechanism in patients undergoing elective open gastric surgery combined with bilateral RSB. The distinctive feature of this trial is the patented technique of catheter insertion during surgical procedures, which is performed under the direct control of the surgeon and entails a continuous infusion of bupivacaine.

## 2. Materials and Methods

The present single-centre randomised controlled trial was conducted at the Riga East Clinical University Hospital (RAKUS), Riga, Latvia. The study design was developed during 2019–2020. The trial protocol and related procedures received approval from the Research Ethics Committee of the University of Latvia (20191202-J02, dated 2 December 2019) and were conducted in accordance with the Declaration of Helsinki (2013). The study protocol was prospectively registered on ClinicalTrials.gov (ID: NCT05592496) in 2022. Participant recruitment and enrollment for the trial took place between October 2022 and September 2023. Written informed consent was obtained from all participants.

### 2.1. Patient Screening

All adult patients aged 18 years and over, who reported at our department were considered eligible for screening. The patients were invited for enrolment if they were indicated to undergo elective open gastric surgery due to malignancy or complicated ulcer disease. Furthermore, we only included patients who were classified as having American Society of Anaesthesiologists (ASA) Grades I, II, or III. Exclusion criteria included incomplete informed consent forms, patients presenting with uncontrolled coagulopathy, allergy to the aesthetic agent, patients undergoing acute gastric surgery, and patients who refused rectus sheath block catheters or continuous anaesthetic infusions.

**Figure 1:**
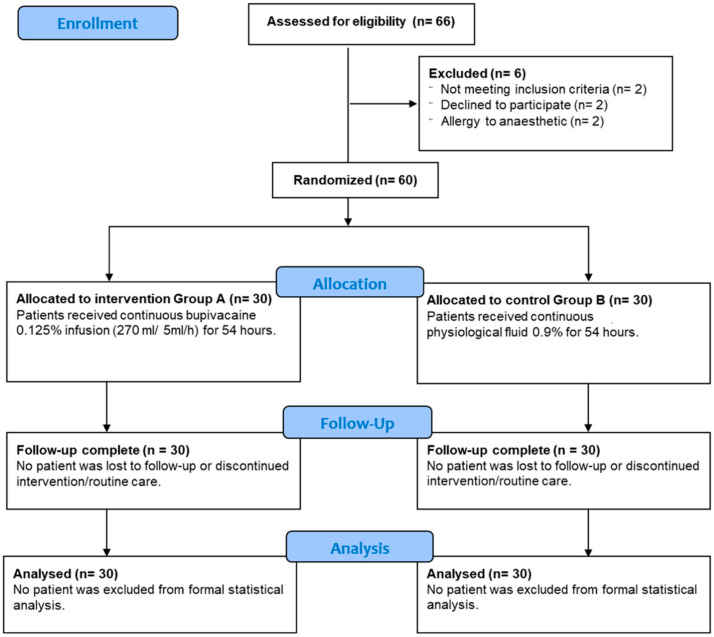
CONSORT flow diagram indicating the screening and enrolment of patients in the present clinical trial.

We also excluded patients who had undergone repeated laparotomy and wound dehiscence since repeated surgical interventions could potentially hinder accurate monitoring of the self-reported pain scale, the quantity and duration of opioids administered, and the secondary outcomes of the study. Accordingly, 66 patients were deemed eligible for participation in the trial. However, six patients were excluded from participation due to conversion to acute surgery post-massive bleeding, bupivacaine allergy, and refusal to participate in the trial (Figure 1).

### 2.2. Randomization and Blinding

Patients were randomly assigned to one of the two study groups—Group A (intervention) and Group B (control). For group allocation, random numbers were generated from 1 to 60 using the SPSS software version 26.0 (IBM SPSS, Inc., Armonk, NY, USA). Patient names were entered in a separate column and were randomly matched with a number using SPSS software. Patients with a number less than 30 were assigned to Group B while those with a number higher than 30 were assigned to Group A. Randomization and patient grouping were carried out by a researcher who did not participate in the surgeries. The patients and the surgical teams were blinded throughout the study, with knowledge of their group allocation restricted to the research team (double-blind study design). Investigators who followed up after surgery were also kept blinded to the group allocation.

### 2.3. Surgical Technique

Before surgery, venous access, pulse oximetry, electrocardiogram, and automated non-invasive arterial blood pressure measurement were performed. General intravenous and endotracheal anaesthesia was performed as usual. The surgical site involved an upper middle laparotomy.

### 2.4. Rectus Sheet Block Technique

**Figure 2:**
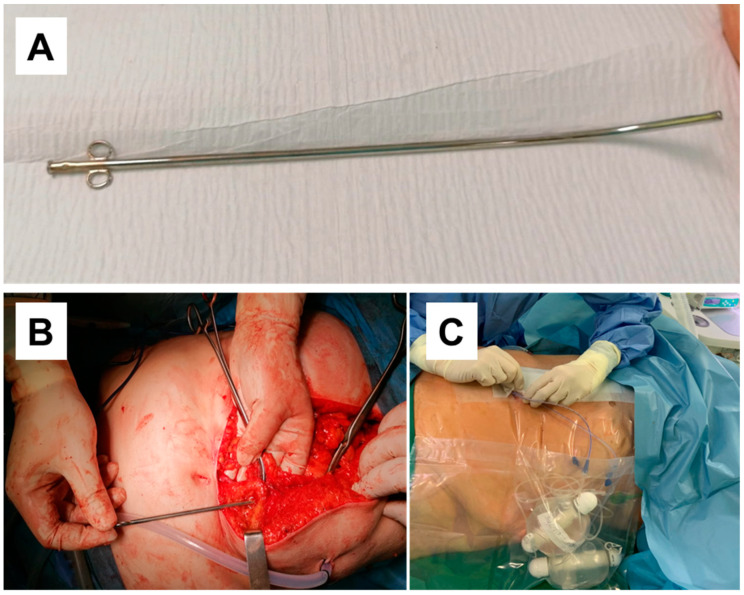
(**A**) Tube for perforated catheter insertion into the retro-muscular space; (**B**) Tube insertion technique; (**C**) Catheters connected to two “Easy Pumps” at the end of the surgery.

Prior to peritoneum closure, once the intra-abdominal stage of the operation (total gastrectomy or gastric resection with Roux-en-Y reconstruction) was complete, the rectus abdominis muscle was identified and palpated. Two perforated catheters with a diameter of 2.7 mm were inserted bilaterally 3–4 cm from the laparotomic wound edges into the retro-muscular plane under the rectus abdominis muscle. Specialised tubes were utilised for the placement of perforated catheters (Figure 2), about 30 min before the end of the surgery. The entire procedure was carried out under the visual control of the operating surgeon.

Following the insertion procedure, the catheters were secured to the subcutaneous tissue with 2-0 Vicryl^®^ sutures. The catheter’s lower portion was then extracted through the laparotomic incision. Subsequently, the aponeurosis was closed with continuous 2-0 Monomax^®^ sutures with the small stitch technique. Cutaneous sutures were placed, and the catheters were positioned alongside the entire surgical incision on both sides (Figure 2). The insertion technique was developed at our institution and in our experience requires approximately one minute to correctly place the catheter in the retro-muscular plane.

Subsequently, two “Easy Pumps” were connected to the retro-muscular catheters by a single individual who was privy to the patient’s group designation. The volume of fluid administered was 270 mL at a rate of 5 mL/h. This comprised 0.125% bupivacaine for Group A and 0.9% NaCl for the Control Group. The patients received a maximum of 288 mg of the bupivacaine solution per day, which was below the recommended upper limit of 400 mg for bupivacaine per day [9]. Infusions were continued for 54 h after which the RSB was removed.

### 2.5. Postoperative Monitoring

After the surgery, patients were shifted and observed in the intermediate care unit. All vital signs were monitored continuously. Upon returning to the ward, patients were allowed to consume water. On the second day, the patients were permitted to consume food in liquid form, and by the third day, they were allowed to ingest solid food. On the first postoperative day, patients were permitted to engage in activities outside of their beds and were provided with a consultation from the physical therapist. On the second postoperative day, patients commenced physical activities in accordance with the recommendations of the physical therapist.

### 2.6. Rescue Analgesia Methods

The pain level was monitored postoperatively using the visual analogue scale (VAS) [3]. VAS is a straight 100 mm line scale, with “no pain” (0 mm) on the left side and the worst pain imaginable (100 mm) on the right side. If the self-reported patient VAS score was >30 mm, Ketorolacum trometamolum 30 mg was injected intravenously. If the VAS score was still more than 30 mm after 30 min, the patient received Trimperidine hidrochloridum 20 mg/1 mL intramuscularly.

### 2.7. Data Collection

The patient’s demographic data, the general performance status according to the ASA classification, the duration of surgery, and the anaesthetics used during surgery were recorded. The primary outcome measures were the quantity of opioid analgesic administered during the initial 72 h postoperative period and the pain level as assessed by the postoperative VAS measurement. Secondary outcomes included the time to the first bowel movements, the patient’s first out-of-bed activity, the duration of hospitalisation, and the incidence of postoperative complications. The quantity of opioid analgesics administered, and the VAS scores were documented at 4, 12, 24, 48, and 72 h postoperatively.

Postoperative complications according to the Clavien–Dindo classification were recorded until the patients were discharged. The transition from a supine to a seated position was designated as an active movement. Additionally, the administration of analgesic drugs and the use of rescue analgesia were documented. Two investigators, who had received sufficient training prior to the study, conducted the postoperative follow-up. They were unaware of which group each patient belonged to.

### 2.8. Statistical Analysis

Statistical analysis was performed using the SPSS software version 22.0 (IBM SPSS, Inc., Armonk, NY, USA). Continuous variable data were expressed as mean and standard deviation (SD) if the data approximated the symmetrical normal distribution. If the data showed skewness, it was described using medians and inter-quartile ranges (IQR). Kolmogorov–Smirnov test was used to evaluate the normal distribution of continuous variables. Normally distributed data comprising continuous variables were analysed using the *t*-test. Skewed distributions were analysed using the Mann–Whitney U test. χ^2^ analysis was used for categorical non-parametric data. The Fisher exact test was used if any cell had an expected value of <5.

## 3. Results

All 60 enrolled patients successfully completed the follow-up and were included in the final analysis. No notable discrepancies were observed between Groups A and B regarding sex, age, height, weight, ASA classification, and duration of surgery (Table 1). Surgically, all RSB blocks were successfully completed, and no complications were observed.

### 3.1. Post-Operative Pain

At all measured time points, VAS scores were significantly lower in interventional Group A compared to the control Group B (Table 2).

### 3.2. Rescue Analgesia Results

In terms of rescue doses for opioids, none of the patients in Group A were indicated for opioid administration per study protocol (Table 3). In Group B, however, patients were provided with Trimperidine hidrochloridum, with the median dosage increasing in the first 24 h after surgery.

The secondary analgesic dose (ketarolacum trometamolum) was not significantly different for the two groups within the first 12 h postoperatively, but the difference was more noticeable 12–48 h after surgery (Table 4).

### 3.3. Time to First Gas and Stool

The time to first gas after surgery in Group A was significantly shorter than in Group B. Furthermore, the first bowel movement occurred two days earlier in Group A than in Group B. The postoperative hospital stay was one day shorter for patients in Group A than for patients in Group B (Table 5).

## 4. Discussion

Open gastrectomy is one of the main procedures that cause severe pain in the postoperative period [10]. Providing adequate pain control after gastrectomies can improve recovery. According to the recommendations outlined in the enhanced recovery after gastrointestinal surgery guidelines, the administration of multimodal opioid-free analgesia and the initiation of early mobilisation represent two key elements of an enhanced recovery protocol following gastrointestinal surgery [11].

The parenteral administration of opioids, including morphine and trimperidine hydrochloride, is an efficacious and preferred approach for providing analgesia. However, the potential for well-documented adverse effects associated with opioids exists, including respiratory depression, nausea, vomiting, sedation, and decreased intestinal motility. Moreover, the prevalence of opioid-induced constipation (OIC) has been documented to range from 15% to 61.7%. The increased use of opioids for non-cancer pain has led to a notable surge in adverse gastrointestinal effects associated with opioids, particularly OIC [12]. Due to alterations in nutritional intake and intestinal flora, OIC in elderly patients can reach up to 80% [13].

Such adverse effects may result in prolonged postoperative hospitalisation, delayed patient recovery, delayed bowel movement, and an increased risk of nosocomial infections [14]. Opioids act on the enteric nervous system by binding to the myenteric and submucosal plexuses, resulting in dysmotility, decreased fluid secretion, sphincter dysfunction, and ultimately opioid-induced bowel dysfunction [15]. Our proposed technique involving RSB with continuous bupivacaine infusions, eliminates the side effects typically associated with parenteral opioids.

In the present study, we conducted a comparative trial between two rather homogeneous cohorts of patients who were indicated to undergo elective open gastrectomies. No statistically significant differences were observed between the two groups with respect to demographic data and operative characteristics. The RSB group that received bupivacaine experienced a significantly lower opioid consumption than the RSB group that did not receive the bupivacaine infusion. The decreased consumption of opioids in the RSB group, in our view, was the primary factor contributing to the reduction in sedation, earlier mobilisation, and improved bowel function.

Furthermore, our technique is advantageous in that a single dose of local anaesthetic may not provide a long-lasting effect [16]. The continuous retro-muscular infusion of local anaesthetic drugs through retro-muscular catheters provides a rather long-lasting and comfortable analgesic effect during the early postoperative period. We believe that a continuous infusion provides enhanced patient outcomes in comparison with a single bolus infusion. In fact, a recent meta-analysis of RSB following abdominal surgery under an ultrasound-guided technique found evidence supporting our hypothesis [17].

The authors noted that a single dose of RSB provided superior pain control only transiently following surgery. Conversely, continuous administration of RSB demonstrated enhanced outcomes in pain management and a reduction in the necessity for opioids on the initial postoperative day [17]. Nonetheless, the results from this evidence synthesis remained inconclusive as many of the included studies found no significant differences in the pain scores between RSB and non-RSB groups.

Another advantage of our technique is the documented antimicrobial efficacy of bupivacaine solution administration in open, elective gastrectomies [13,18,19]. As observed also in our cohort, no patients developed wound infections. It is beyond dispute that the use of continuous bupivacaine infusion through catheters inserted in the retro-muscular rectus sheath space is a well-established method of analgesia [20]. The innovation presented in this study is the technique used for the placement of the catheters. The use of an ultrasound device is not required. Furthermore, the procedure for precise catheter placement is considerably more expeditious, requiring only approximately 1–2 min. No additional specialist input is required for this procedure.

Nonetheless, our study leaves room for further augmentation and improvements. For example, we conducted pain assessments using the visual analogue scale (VAS). It would be preferable to use an objective pain assessment instrument, such as the ANI device, which quantifies the Analgesia Nociception Index. Additionally, the plasma concentration of bupivacaine was not measured, which could be employed to more accurately regulate the level of analgesia. In conclusion, the present study demonstrated the convenience and reliability of open RSB catheter placement, which resulted in a positive analgesic effect of bupivacaine for continuous wound infiltration through catheters placed in the rectus sheath.

## 5. Conclusions

The use of a bilateral RSB with a continuous bupivacaine infusion can facilitate the recuperation of gastrointestinal function and reduce the duration of hospitalisation for patients undergoing open upper gastrointestinal surgery. The procedure involves a markedly reduced use of opioids, unsuppressed normal intestinal function, and precise local infiltration anaesthesia placement. Our novel approach to RSB catheter placement allows for precise localization in a few minutes without the necessity for specialised equipment. We advise to exercise caution when employing the procedure on patients who have previously undergone laparotomies with extended midline incisions, particularly on the first postoperative day.

## Figures and Tables

**Table 1:** Demographic and surgical characteristics of the patients in this study.

Characteristic	Group A (*n* = 30)	Group B (*n* = 30)	*p* Value
Sex (*n*, %)			
Male	16 (53%)	16 (53%)	1.000 *
Female	14 (47%)	14 (47%)	
Age, years (median, IQR)	71 (63 to 83)	68 (57 to 74)	0.034 **
BMI, kg/m^2^ (mean, SD)	25.1 (5.3)	26.6 (4.2)	0.242 ***
ASA Grade (*n*, %)			
ASA I	13 (43%)	11 (37%)	0.558 *
ASAII	10 (33%)	14 (47%)	
ASA III	7 (24%)	5 (16%)	
Duration of surgery, min (median, IQR)	180 (160 to 205)	188 (160 to 250)	0.510 **

* Chi-square χ^2^ test. ** Mann–Whitney U test. *** Independent samples *t*-test.

**Table 2:** Postoperative visual analogue pain scores in mm at rest (median, IQR).

Time After Surgery	Group A (*n* = 30)	Group B (*n* = 30)	*p* Value *
4 h	40 (30 to 60)	80 (80 to 90)	<0.001
12 h	40 (20 to 40)	70 (70 to 80)	<0.001
24 h	20 (18 to 30)	70 (60 to 70)	<0.001
48 h	10 (10 to 20)	60 (50 to 70)	<0.001

* Mann–Whitney U test.

**Table 3:** Postoperative opioid analgesia dosage of Trimperidine hidrochloridum, mg (median, IQR).

Time After Surgery	Group A (*n* = 30)	Group B (*n* = 30)
0–4 h	-	20 (0 to 20)
4–12 h	-	40 (40 to 60)
12–24 h	-	60 (40 to 60)
24–48 h	-	40 (40 to 40)

**Table 4:** Postoperative NSAID analgesia dosage of Ketarolacum trometamolum, mg (median, IQR).

Time After Surgery	Group A (*n* = 30)	Group B (*n* = 30)	*p* Value *
0–4 h	30 (30 to 30)	30 (30 to 30)	0.054
4–12 h	30 (0 to 30)	60 (60 to 60)	<0.001
12–24 h	-	60 (60 to 60)	-
24–48 h	-	60 (60 to 60)	-

* Mann–Whitney U test.

**Table 5:** The recovery time postoperatively after upper gastrointestinal surgery (median, IQR).

Characteristic	Group A (*n* = 30)	Group B (*n* = 30)	*p*-Value *
First gas (post op. day)	2 (2 to 3)	3 (3 to 3)	0.001
First stool (post op. day)	3 (3 to 4)	5 (4 to 5)	<0.001
Postoperative days	6 (5 to 7)	7 (6 to 10)	<0.001
First out-of-bed activity	3 (2 to 3)	4 (3 to 5)	<0.001

* Mann–Whitney U test.

## Data Availability

The data are available from the corresponding author on reasonable request.
