# Correction: Opincans et al. Bilateral Rectus Sheath Block with Continuous Bupivacaine Infusions After Elective Open Gastrectomy: A Randomized Controlled Trial. *Medicina* 2024, *60*, 1992

**DOI:** 10.3390/medicina62071262

**Published:** 2026-06-30

**Authors:** Janis Opincans, Igors Ivanovs, Aleksejs Miscuks, Janis Pavulans, Katrina Deja Martinsone, Agris Rudzats, Zurabs Kecbaja, Olegs Gutnikovs, Aleksejs Kaminskis

**Affiliations:** 1 Department of Surgery, Riga East Clinical University Hospital, 1038 Riga, Latvia; 2 Faculty of Medicine, University of Latvia, 1004 Riga, Latvia; 3 Faculty of Medicine, Riga Stradins University, 1007 Riga, Latvia

**Text Correction**

There was an error in the original publication [1]. There is a discrepancy between the ClinicalTrials registration date and the beginning of the patient enrollment. A correction has been made to the first paragraph of section Materials and Methods:

The present single-centre randomised controlled trial was conducted at the Riga East Clinical University Hospital (RAKUS), Riga, Latvia. The study design was developed during 2019–2020. The trial protocol and related procedures received approval from the Research Ethics Committee of the University of Latvia (20191202-J02, dated 2 December 2019) and were conducted in accordance with the Declaration of Helsinki (2013). The study protocol was prospectively registered on ClinicalTrials.gov (ID: NCT05592496) in 2022. Participant recruitment and enrollment for the trial took place between October 2022 and September 2023. Written informed consent was obtained from all participants.

The authors state that the scientific conclusions are unaffected. This correction was approved by the Academic Editor. The original publication has also been updated.
